# Functional Properties of Food Origin *Lactobacillus* in the Gastrointestinal Ecosystem—In Vitro Study

**DOI:** 10.1007/s12602-018-9458-z

**Published:** 2018-08-23

**Authors:** Dorota Zielińska, Ewa Długosz, Anna Zawistowska-Deniziak

**Affiliations:** 1 Department of Food Gastronomy and Food Hygiene, Faculty of Human Nutrition and Consumer Sciences, Warsaw University of Life Science – SGGW, Nowoursynowska 159, 02-776 Warsaw, Poland; 2 Department of Preclinical Sciences, Faculty of Veterinary Medicine, Warsaw University of Life Science – SGGW, Nowoursynowska 159, 02-776 Warsaw, Poland; 3 Witold Stefański Institute of Parasitology, Polish Academy of Sciences, Twarda 51/55, 00-818 Warsaw, Poland

**Keywords:** *Lactobacillus*, Probiotic, Cytokine, Apoptosis, Caco-2 cells, In vitro study

## Abstract

In the current study, the probiotic activity of ten *Lactobacillus* (*Lb*.) strains, previously isolated from the traditional Polish fermented vegetable, was characterized. Strains were assessed for adhesion to human intestinal epithelial Caco-2 cells and regulation of selected cytokine production (IL-1β, IL-6, IL-10, IL-23, and TNF-α) by THP-1 macrophages. The effect of tested strains on Caco-2 cell apoptosis was also investigated using a caspase-3 assay. Adhesion capacity was strain-dependent (1.29–8.24% of initial population). Highest adhesion was observed for *Lb*. *brevis* O24. All *Lactobacillus* strains investigated in this study stimulated two- to threefold increase in TNF-α, IL-1β, and IL-6 production, compared to the control. Additionally, selected strains of *Lactobacillus* caused a significant decrease of pro-inflammatory cytokine production by lipopolysaccharide (LPS)-stimulated THP-1 cells. Almost all *Lactobacillus* investigated in this study are potent stimulators of IL-10 production. All tested *Lactobacillus* cells slightly increased the caspase-3 activity in Caco-2 cells. *Lb*. *casei* O18 was the most inducing strain. The tested strains had no effect on staurosporine (STS)-induced caspase-3 activity. According to these results, the most promising strains are *Lb*. *plantarum* O20, two strains *Lb*. *brevis* O22 and O24, and *Lb*. *rhamnosus* K3. These newly identified lactobacilli hold promise for use as probiotics in functional food applications.

## Introduction

The genus *Lactobacillus* (*Lb*.) comprises a large heterogeneous group of low-G+C gram-positive, nonsporulating, rod-shaped, and facultative anaerobic bacteria. Taxonomically, the genus *Lactobacillus* belongs to the phylum Firmicutes, class Bacilli, order Lactobacillales, family Lactobacillaceae [1]. Currently, the genus *Lactobacillus* comprises more than 200 species enlisted in LPSN database (The List of Prokaryotic Names with Standing in Nomenclature, http://www.bacterio.net). The lactobacilli occupy a variety of niches including milk and plant surfaces, as well as the gastrointestinal tract of humans and animals. Humans have safely been ingesting lactobacilli for centuries in fermented foods and beverages, and more recently, in probiotic products. This makes lactobacilli an interesting microbial group, whose population can be made up by transient members or bystanders of the gastrointestinal tract. *Lactobacillus* bacteria play a crucial role in maintaining the ecological equilibrium of these environments, through direct antimicrobial effects, the enhancement of mucosal barrier integrity, and the immune modulation [2–4].

One of the most promising areas of development in the human nutritional field over the last two decades has been the use of probiotics and recognition of their role in human health and disease. The means by which probiotic bacteria elicit their health effects are not understood fully, but may include competitive exclusion of enteric pathogens, neutralization of dietary carcinogens, production of antimicrobial metabolites, and modulation of mucosal and systemic immune functions [5]. According to the currently adopted definition by the Food and Agriculture Organization/World Health Organization [6], probiotics are defined as “live microorganisms which when administered in adequate amounts confer a health benefit on the host.” However, in recognition of the increasing evidence for effects of probiotics on immune function, this definition has sometimes been extended to “live microorganisms, that when included in foods can influence the composition and activity of the gut microbiota, modulate the inflammatory response, improve the nonspecific intestinal barrier, and reinforce or modulate the mucosal and the systemic immune responses” [7].

In addition to the effects of probiotics on nonimmunologic gut defense, which is characterized by stabilization of the gut microflora, probiotic bacteria are shown to promote the endogenous host defense mechanisms [8]. Moreover, these bacteria have been shown to stimulate nonspecific host resistance to microbial pathogens, and thereby aid in immune elimination, and to modulate the host immune responses to potentially harmful antigens with a potential to downregulate hypersensitivity reactions [9, 10].

The functionality of probiotics is generally strain-dependent. Often, strains used as probiotics are derived from human sources, but this is not a requirement. Selection of probiotics involves screening for safe nonpathogenic bacteria, which are evaluated on the basic features, including acid and bile resistance and ability to adhere to gut epithelial cells. The next step in functionality assessment includes characterization of core health benefits [6].

Recently, some studies have reported that *Lactobacillus* isolated from unconventional sources possesses selected beneficial features [11]. Several lactic acid bacteria from fermented food have also been isolated in our laboratory and screened for probiotic properties. In a previous study [12], fourteen *Lactobacillus* strains were isolated from pickled vegetables and characterized using phenotypic and genotypic methods. Moreover, their in vitro resistance to gastrointestinal conditions, antibiotic resistance, and enzymatic profiles were investigated. Ten of the selected *Lactobacillus* strains were shown to be safe and survive under gastrointestinal conditions, what promotes them for future in vitro and in vivo studies.

The objective of the current study was to investigate the probiotic activity of ten previously isolated lactobacilli from traditional Polish fermented vegetables in terms of their ability to adhere to enterocytes and the influence on host immune response and apoptosis of host cells. The results of these tests will allow the evaluation of these strains as potential probiotics with immunomodulatory properties for human use.

## Material and Methods

### Bacterial Strains and Growth Condition

Ten *Lactobacillus* strains were used: *Lb*. *casei* (O12, O13, O16, O18); *Lb*. *plantarum* (O19, O20); *Lb*. *brevis* (O22, O24); *Lb*. *rhamnosus* K3; and *Lb*. *johnsonii* K4. The strains were isolated from a traditional fermented cabbage and cucumber from Poland and identified by molecular typing and selected in vitro probiotic properties as described previously [12]. The well-studied commercial probiotic strains *Lb*. *plantarum* 299v and *Lb*. *rhamnosus* GG were used as reference strains. Lactobacilli strains were propagated in de Man, Rogosa, and Sharpe (MRS, Merck, Poland) broth. Organisms were subcultured every 20 h or 24 h twice before experimental use. Bacterial strains were maintained in a mixture of MRS broth (800 μl) and glycerol (200 μl) at − 80 °C until further use.

The colony-forming units (CFU)/ml were determined by plating serial tenfold dilutions in sterile peptone saline (Noack, Poland) on MRS (Merck, Poland) agar, for the enumeration of *Lactobacillus* strain. The plates were incubated at anaerobic conditions for 48 h at 37 °C.

### In Vitro Adhesion Assay

Caco-2 epithelial cells, originating from human colorectal adenocarcinoma (ATCC HTB-37), were used in their terminally differentiated state to mimic small intestine mature enterocytes. The cells were cultured in Dulbecco’s modified Eagle’s minimal essential medium (DMEM, Biowest) supplemented with 10% (*v*/*v*) heat-inactivated fetal bovine serum (FBS) (Biowest), 2 mM l-glutamine (Biowest), 100 U/ml penicillin, and 100 mg/ml streptomycin (Biowest) at 37 °C in an atmosphere containing 5% CO_2_. The culture medium was changed every other day and replaced by fresh DMEM. Cells were used between 26 and 40 cell passages.

The adherence of the LAB strains to the Caco-2 cell line was tested according to [13] with slight modifications. Caco-2 was seeded in 24-well tissue culture plates (NUNC) at 5 × 10^5^ cells per well and grown during 14 days to obtain a monolayer of differentiated and polarized cells. The culture medium was changed every 2 days. Bacterial cultures were grown for 24 h at 37 °C, and they were sedimented by centrifugation, 10,000×*g* for 10 min. Then, they were washed twice, resuspended in fresh DMEM, and an inoculum of 10^8^ CFU was added to each well of the tissue culture plate containing Caco-2 monolayer in a final volume of 1 ml. The plates were incubated for 2 h at 37 °C in a 5% CO_2_ atmosphere. After incubation, unattached bacteria were removed by washing the monolayers four times with sterile PBS. Additionally, 200 μl of trypsin (2.5%, *w*/*v*) was added to detach the monolayer. The adhered bacteria were incubated for 15 min at room temperature and recovered by repeated pipetting with 800 μl of sterile ultrapure water and enumerated by plating serial dilutions on MRS agar. Adhesion values (%) were calculated as follows:

$$ \%\mathrm{Adhesion}=\left({V}_1\times 100\right)/{V}_0 $$

where *V*_0_ is the initial viable count of bacteria tested, and *V*_1_ is the viable bacteria count obtained from the Caco-2 cells, at the end of the experiment.

Each adhesion assay was conducted in triplicate.

### Cytokine Quantification (ELISA)

THP-1 monocytes were purchased from ATCC. Cells were cultured in complete medium (CM) which consisted of RPMI 1640 medium (Sigma–Aldrich) with glutamine (2 mM), buffered with NaHCO_3_, (without HEPES) supplemented with 10% heat-activated FBS (Sigma–Aldrich), and 100 U/ml penicillin + 100 μg/ml streptomycin (Sigma–Aldrich). When appropriate cell numbers were achieved, cells were seeded in 24-well plates at 1 × 10^6^ cells/ml. In order to differentiate THP-1 monocytes to macrophages, cells were seeded in 24-deep-well dishes in 1 ml of CM, treated with phorbol 12-myristate 13-acetate (PMA) (Sigma–Aldrich) (30 ng/ml), and incubated for 48 h. After this time, cells were adherent. Macrophages were rinsed three times with prewarmed RPMI 1640, and 1 ml of CM was added to each well. In parallel, in order to induce pro-inflammatory conditions in the culture bacterial, lipopolysaccharide (LPS from *Escherichia coli* O127:B8, Sigma–Aldrich) (100 ng/ml) was added. Cell cultures without LPS stimulation served as controls. After 6 h of treatment with *Lactobacillus* bacteria (inoculum of 10^8^ CFU/ml), the levels of cytokines (IL-1β, IL-6, IL-10, IL-23, and TNF-α) produced by macrophages were measured in the culture supernatant using Opti EIA ELISA Set (BD). The results were read on a BioTek Synergy HT microplate reader, and cytokine concentrations were calculated using standard curves according to manufacturer’s protocols. Each assay was performed in triplicate.

### Caco-2 Cell Apoptosis—Caspase-3 Assay

Apoptosis in Caco-2 cells was measured using a Caspase-3/CPP32 Colorimetric Protease Kit (Invitrogen). Measurement of caspase-3 activity was based on spectrophotometric detection of the chromophore p-nitroaniline (*p*NA) after cleavage from the labeled substrate DEVD-*p*NA. The *p*NA absorbance can be quantified. The assay was performed according to producer manual protocol. Briefly, Caco-2 was seeded in 24-well tissue culture plates at 5 × 10^5^ cells per well and grown during 14 days to obtain a monolayer of differentiated and polarized cells. Overnight, *Lactobacillus* cultures were centrifuged, washed twice with PBS, and suspended in DMEM so that each well contained 10^8^ CFU bacterial cells per 1 ml of culture medium. Caco-2 cells were treated with staurosporine (STS; Sigma–Aldrich; concentration 2 μM) resuspended in DMSO, to induce apoptosis. Concurrently, control cultures without induction were prepared. The plates were incubated for 4 h at 37 °C in a 5% CO_2_ atmosphere. After incubation, a caspase-3 kit was implemented, and the absorbance of *p*NA from apoptotic samples and un-induced control samples was detected at 405 nm in an MRX microplate reader (Dynatech Laboratories).

### Statistical Analysis

Data are expressed as mean ± standard error (SE) calculated over three independent experiments performed in triplicate. Analysis of statistical significance was performed with nonparametric Mann–Whitney test. A value of *P* < 0.05 was considered to be significant. Analysis was done using Statgraphics Plus 4.1 software.

## Results

### In Vitro Adhesion Assay

The adhesion of ten selected *Lactobacillus* strains to Caco-2 cells in comparison with reference probiotic strains is shown in Table 1. The initial *Lactobacillus* cell count in all cases was 1.62–3.87 × 10^8^ CFU/ml, and after incubation, at the end of experiment, 4.20 × 10^6^–2.05 × 10^7^ bacteria cells adhered to Caco-2 cells were recovered, that corresponded to 1.29–8.24% of initial population. Adhesion capacity was strain-dependent. Highest adhesion of 8.24% was observed for *Lb*. *brevis* O24.

**Table 1:** The bacterial adhesion capacity of *Lactobacillus* strains to Caco-2 cells

Strains used in the study		Initial count of bacteria [CFU/ml]	Adhered count of bacteria [CFU/ml]	Adhesion A [%]
*Lactobacillus casei*	O12	2.41 ± 1.25 × 10^8^	1.56 ± 1.13 × 10^7^	6.47 ± 0.21
*Lactobacillus casei*	O13	3.87 ± 0.87 × 10^8^	1.02 ± 0.96 × 10^7^	2.64 ± 0.09
*Lactobacillus casei*	O16	2.14 ± 1.05 × 10^8^	1.59 ± 0.93 × 10^7^	7.43 ± 0.15
*Lactobacillus casei*	O18	3.52 ± 1.20 × 10^8^	2.05 ± 1.07 × 10^7^	5.82 ± 0.17
*Lactobacillus plantarum*	O19	3.25 ± 0.65 × 10^8^	4.20 ± 1.32 × 10^6^	1.29 ± 0.03
*Lactobacillus plantarum*	O20	3.69 ± 0.74 × 10^8^	1.01 ± 0.94 × 10^7^	2.74 ± 0.06
*Lactobacillus brevis*	O22	3.60 ± 0.98 × 10^8^	1.48 ± 0.22 × 10^7^	4.11 ± 0.11
*Lactobacillus brevis*	O24	2.05 ± 1.35 × 10^8^	1.69 ± 0.68 × 10^7^	8.24 ± 0.23
*Lactobacillus rhamnosus*	K3	2.76 ± 1.09 × 10^8^	1.13 ± 0.31 × 10^7^	4.09 ± 0.04
*Lactobacillus johnsonii*	K4	1.62 ± 0.87 × 10^8^	3.20 ± 0.95 × 10^6^	1.98 ± 0.03
*Lactobacillus plantarum*	299v	2.31 ± 0.98 × 10^8^	9.20 ± 1.16 × 10^6^	3.98 ± 0.08
*Lactobacillus rhamnosus*	GG	2.41 ± 0.86 × 10^8^	9.30 ± 0.88 × 10^6^	3.86 ± 0.05

### Cytokine Quantification

**Fig. 1:**
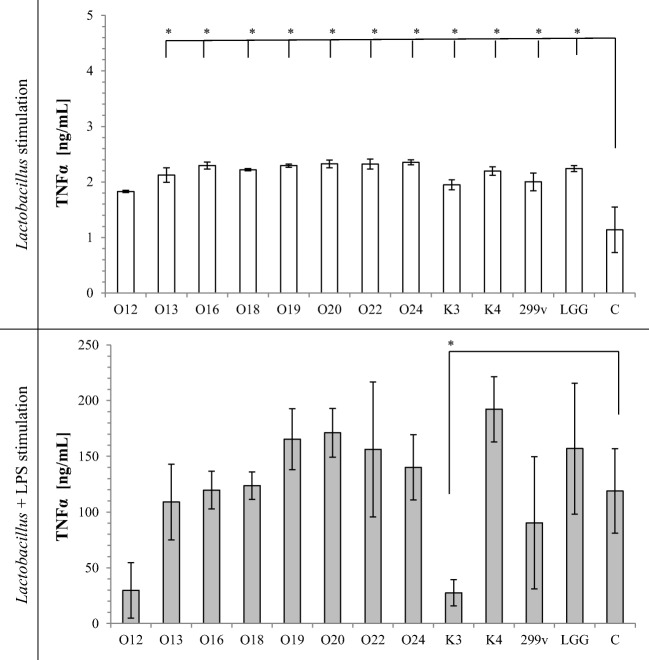
Effect of *Lactobacillus* strains on the production of TNF-α in THP-1 cells. The results are expressed as mean ± SD, *n* = 3; *P < 0.05 compared to control sample—C

**Fig. 2:**
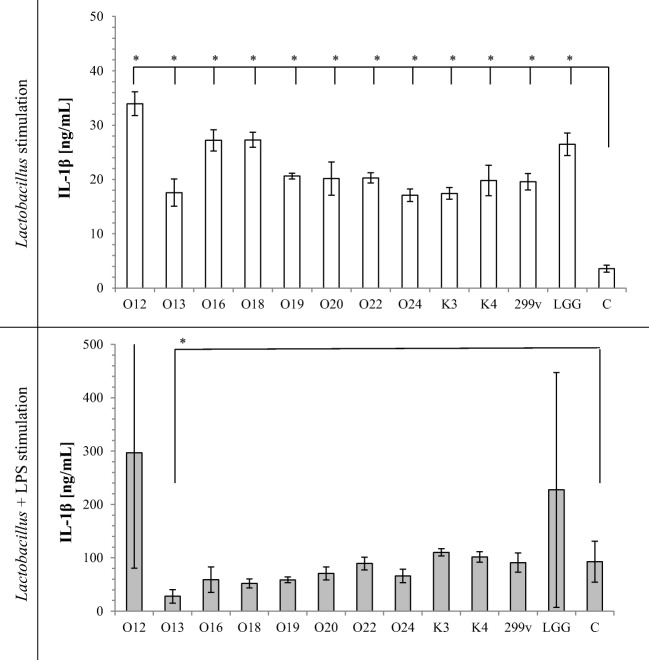
Effect of *Lactobacillus* strains on the production of IL-1β in THP-1 cells. The results are expressed as mean ± SD, *n* = 3; *P < 0.05 compared to control sample—C

**Fig. 3:**
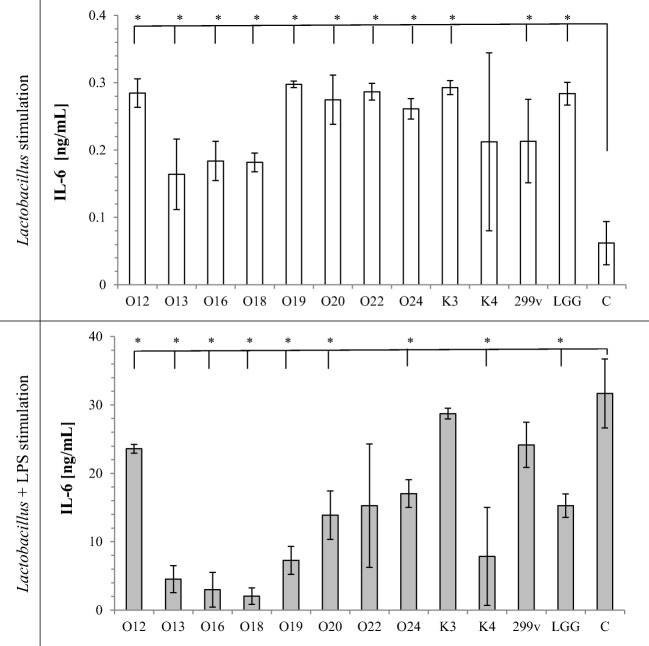
Effect of *Lactobacillus* strains on the production of IL-6 in THP-1 cells. The results are expressed as mean ± SD, *n* = 3; *P < 0.05 compared to control sample—C

TNF-α secretion by macrophages induced with *Lactobacillus* cells alone or *Lactobacillus* cells with LPS stimulation is shown on Fig. 1. Almost all tested *Lactobacillus* strain cells significantly increased TNF-α secretion in comparison to sample containing only THP-1 cells in RPMI medium. *Lb*. *casei* O12 did not induce TNF-α secretion alone. Tested strains had different effects on LPS-mediated activation of macrophages. Exposure of LPS-stimulated macrophages to viable bacteria did not induce TNF-α secretion. Moreover, *Lb*. *rhamnosus* K3 strain significantly decreased LPS-induced TNF-α secretion. Similarly, IL-1β secretion (Fig. 2) induced with *Lactobacillus* cells alone increased, whereas LPS-stimulated THP-1 cells did not change IL-1β secretion, except *Lb*. *casei* O13, which significantly decreased IL-1β secretion. All the tested strains, except *Lb*. *johnsonii* K4, used alone as stimulators, increased production of IL-6 in THP-1 cells (Fig. 3). On the other hand, LPS-activated THP-1 macrophages induced with *Lactobacillus* cells decreased IL-6 production (except *Lb*. *rhamnosus* K3 and *Lb*. *plantarum* 299v), in comparison to control sample. Stimulation of THP-1 macrophages with *Lb*. *casei* O12, *Lb*. *rhamnosus* K3, and *Lb*. *plantarum* 299v cells alone significantly increased secretion of IL-23, whereas other used strains significantly decreased IL-23 secretion. Only *Lb*. *rhamnosus* GG had no influence on IL-23 production. In comparison, LPS-activated THP-1 macrophages induced with *Lactobacillus* cells decreased IL-23 secretion in almost all samples, except *Lb*. *rhamnosus* K3 and *Lb*. *johnsonii* K4; however, these differences were not statistically significant. Production of the anti-inflammatory IL-10 was strain-dependent. Almost all *Lactobacillus* strains increased IL-10 secretion alone (except two strains of *Lb*. *casei* O13 and O16). After LPS stimulation of macrophages, *Lb*. *plantarum* O20 and two strains *Lb*. *brevis* O22 and O24 significantly increased, whereas *Lb*. *casei* O13, O16, and O18 significantly decreased IL-10 secretion, in comparison to control.

### Caco-2 Cell Apoptosis—Caspase-3 Assay

To assess the effects of *Lactobacillus* and/or STS stimulation, caspase-3 activation after inoculation was determined (Fig. 6). All tested *Lactobacillus* cells increased the caspase-3 activity after 4 h of inoculation, compared to DMSO control sample. *Lb*. *casei* O18 was the most inducing strain. In STS-stimulated samples, *Lactobacillus* cells did not significantly change caspase-3 activity comparing to cells treated with STS alone. Only *Lb*. *casei* O13 significantly increased caspase-3 activity.

## Discussion

The ten tested strains were found to possess desirable probiotic properties, as demonstrated in a series of established in vitro tests [12]. It was proven that the selected *Lactobacillus* cells are safe and can survive the gastrointestinal stress conditions. However, to confirm their probiotic action, more experimental data are required. We investigated the adhesion of food origin *Lactobacillus* to Caco-2 cells, compared to the reference strains. The ability to adhere to intestinal epithelium is considered an important selection criterion for bacteria intended for probiotic use and to understand more about the complex interaction between probiotic and the host.

Human enterocyte-like Caco-2 cells have often been used for in vitro studies on the mechanisms of cellular adhesion of lactobacilli. The Caco-2 cells line has been used as an in vitro model for intestinal epithelium, because it has been reported to have a good correlation to in vivo results [14–16].

In our study, probiotic adhesion differed depending on *Lactobacillus* strain and species, what was in agreement with other research [17–19]. We have considered the tested *Lactobacillus* strains as able to bind to Caco-2 cells, with adhesion capacity varying from 1.98 to 8.24%. Six of the tested strains (O12, O16, O18, O22, O24, and K3) had higher adhesion capacity than referenced probiotic strains used in this study. It has been reported that various *Lactobacillus* probiotic strain adhesion to Caco-2 cells ranged between 2 and 14% and the phenomenon was strain-dependent [20, 21]. The adhesion percentage of *Lb*. *rhamnosus* GG and *Lb*. *plantarum* 299v which were used as positive controls was similar to those obtained with the same strains in other studies [19, 22, 23].

Several in vitro studies suggested that probiotic adhesion may be cell surface hydrophobicity dependent [24]. Hydrophobicity may facilitate the first contact between the microorganism and the host cell. However, this nonspecific initial interaction is generally considered as weak and reversible. Bacterial adhesion mechanism involves diverse surface molecules such as lipoteichoic acids and proteinaceous compounds as main mediators of bacterial attachment to epithelial cell lines [25]. Some authors have suggested that these surface properties correlate with their adhesive capacity [26, 27]. On the other hand, in many studies, it was proven that there was no correlation between hydrophobicity and bacterial adhesion [22, 28, 29]. Our calculation on the basis of the previous study [12] also confirms these findings. However, other authors observed that selected *Lactobacillus* strains had relatively high hydrophobicity along with relatively high autoaggregation and adhesion ability, which suggest their potential immunomodulatory activity in the GI tract [30].

**Fig. 4:**
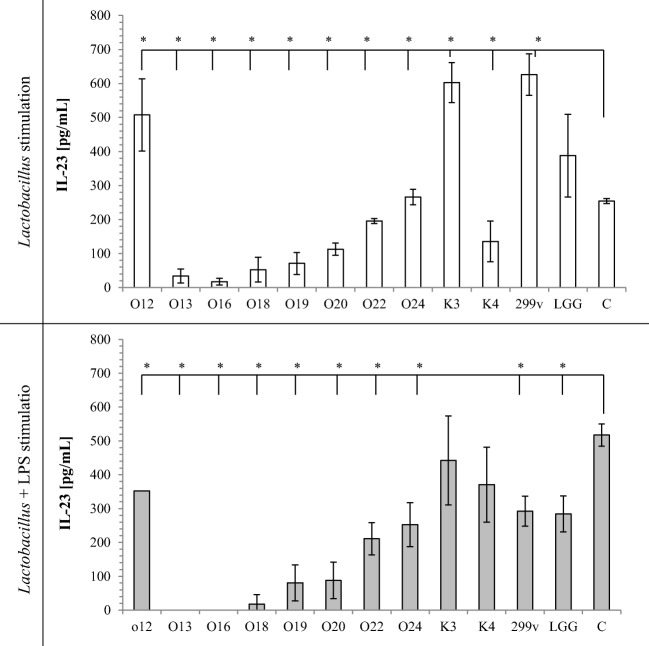
Effect of *Lactobacillus* strains on the production of IL-23 in THP-1 cells. The results are expressed as mean ± SD, *n* = 3; *P < 0.05 compared to control sample—C

**Fig. 5:**
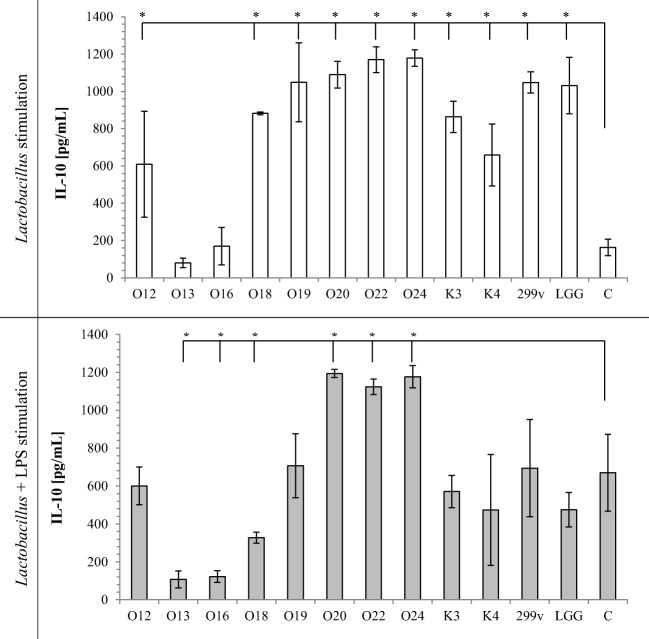
Effect of *Lactobacillus* strains on the production of IL-10 in THP-1 cells. The results are expressed as mean ± SD, *n* = 3; *P < 0.05 compared to control sample—C

It has been suggested that the adhesion capacity may be correlated with transient colonization of intestinal cells, which could be a factor for their immunomodulation by probiotics. The potential immune-stimulating properties of *Lactobacillus* have attracted attention in recent years [10, 31]. *Lactobacillus* cells can elicit innate and adaptive immune responses in the host by binding to pattern recognition receptors (PRR) expressed on cells of the human host. PRR recognize conserved bacterial molecular structures microbe-associated molecular patterns (MAMPs) and signal to induce the production of cytokines, chemocytokines, and other innate effectors [4]. In vitro assays with different types of immune cells, such as human monocyte-derived, human peripheral blood mononuclear cells (PBMC), and mouse bone marrow-derived cells, have often been used to assess the immunomodulatory potential of different species of bacteria. In our investigation, we used THP-1 monocytic cell line for testing the interaction between *Lactobacillus* strains and the immune system. All *Lactobacillus* strains investigated in this study stimulated two- to threefold increase in TNF-α, IL-1β, and IL-6 production, compared to the control. Secretion of IL-23 and IL-10 changed depending on the strain used. Basic cytokine secretion from THP-1 cells is low, often below the detection limit. Cytokine secretion responses noticed in our study are typical for bacteria presence and in accordance with other studies [32, 33]. TNF-α, IL-1β, IL-23, and IL-6 are pro-inflammatory cytokines, which are produced by the host in response to bacterial colonization or invasion and hence are central to the host defense mechanism against pathogens [34]. Lipopolysaccharides from gram-negative bacteria are known as stimulators of a range of cells inducing the synthesis of cytokines. LPS-induced THP-1 cell secretion of TNF-α, IL-1β, and IL-6 cytokines was 100-fold higher comparing to controls (see Figs. 1, 2, and 3) but only twofold higher in case of IL-23 (Fig. 4) and IL-10 (Fig. 5). However, in all cases, addition of *Lactobacillus* cells did not increase TNF-α, IL-1β, IL-23, and IL-6 cytokine production in comparison to control. Moreover, tested strains suppressed IL-23 and IL-6 production by LPS-stimulated THP macrophages. It should be noted here that IL-6 is known to have both pro- and anti-inflammatory properties. It has a very important role in resolving of the initial inflammatory reaction. The presence of IL-6 may help to convert an innate response to a more specific and sustained adaptive response against pathogens [35].

IL-10 is an anti-inflammatory cytokine, which downregulates antigen presentation and inhibits the activation of macrophages. Moreover, IL-10 suppresses IL-12 production and consequently, IFN-gamma production [4]. Hence, almost all *Lactobacillus* investigated in this study are potent stimulators, with *Lb*. *plantarum* O20 and *Lb*. *brevis* O22 and O24 strains being the strongest IL-10 cytokine inducers and *Lb*. *casei* O13 and O16 being the lowest IL-10 stimulators. Generally, *Lb*. *casei* species were the weakest, whereas *Lb*. *plantarum* and *Lb*. *brevis* species were the strongest IL-10 stimulators.

Probiotics can improve intestinal host defenses not only by normalizing intestinal colonization patterns but also by directly affecting intestinal epithelial function such as cytokine secretion, inflammation, and regulation homeostatic processes such as apoptosis [36]. Apoptosis is a cell suicide mechanism to control cell numbers in tissues and eliminate individual cells. However, unscheduled apoptosis of certain cells can be detrimental [37]. Caspase-3 is a key executioner caspase in the proteolytic cascade that leads to apoptotic cell death, and cleaves a number of structural proteins during the execution phase of apoptosis.

**Fig. 6:**
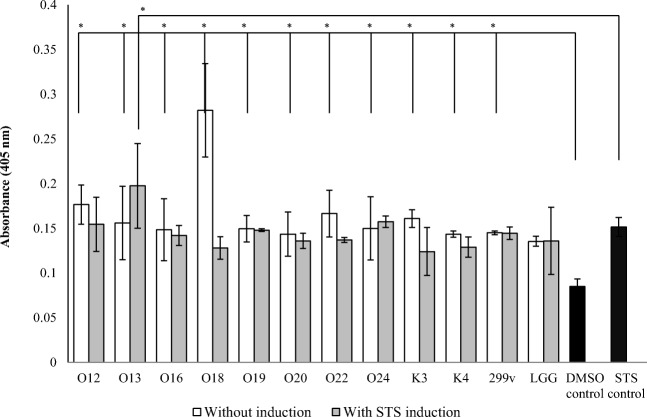
Effect of *Lactobacillus* strains on the activity of caspase-3 in Caco-2 cells. The results are expressed as mean ± SD, *n* = 3; *P < 0.05 compared to control samples

We have observed that *Lactobacillus*-induced apoptosis was accompanied by changes in caspase-3 activity. The caspase-3 activity increased after 4 h of incubation. The most inducing strain was *Lb*. *casei* O18. This indicated that tested *Lactobacillus* cells can slightly induce apoptosis in Caco-2 cells, what is with agreement with other findings (Fig. 6). Apoptosis is implicated in the generation and resolution of inflammation in response to bacterial cells. All gram-negative bacterial pathogens produce lipoproteins, such as LPS, which trigger the innate immune response. Strains of lactobacilli differ considerably in their ability to trigger immune signaling, which is related to the chemical structure of lipoteichoic acid and the abundance of lipoproteins [10]. Although many bacterial pathogens induce apoptosis in host cells, the implications of this phenomenon remain elusive. Bacterial lipoprotein-induced apoptosis could be important for the initiation of inflammation, the resolution of inflammation, and the generation of the proper signals necessary for adaptive immune responses [38].

On the other hand, tested *Lactobacillus* strains do not increase chemically (STS) induced apoptosis; moreover, nine of them (*Lb*. *casei* O16 and O18; *Lb*. *plantarum* O19, O20, and 299v; *Lb*. *brevis* O22; *Lb*. *rhamnosus* K3 and GG; and *Lb*. *johnsonii* K4) decreased caspase-3 activity after STS stimulation, but the changes were not significant.

Many authors indicated that probiotics have the ability to prevent inflammation-induced and chemical-induced cell apoptosis. It has been reported that *Lb*. *acidophilus* ATCC 4356S-layer proteins protected against *Salmonella*-induced apoptosis through reduced caspase-3 activation [39]. Other authors [40] reported that *Lb*. *rhamnosus* GG was able to prevent cytokines from inducing apoptosis by activating anti-apoptotic genes (Akt/protein kinase B) and inhibiting activation of pro-apoptotic genes (p38/MAP-kinase), in both mouse and human colonic epithelial cells. The same group later [41] demonstrated that soluble proteins, p75 and p40, from LGG mediate the cytoprotective effects, highlighting the potential benefit of bacterial products rather than bacteria itself.

Also, effect of probiotic *Lb*. *plantarum* 299v on cytokine-induced apoptosis was evaluated [42]. The authors demonstrated that cell pre-incubation with *Lb*. *plantarum* 299v reduced HT-29 caspase+ cells, suggesting that the reduction of caspase-dependent programmed cell death may be mediated by caspase-3 inhibition.

Moreover, it has been reported that in vitro *Lb*. *rhamnosus* GG can reduce intestinal epithelial apoptosis stimulated with a broad-spectrum pro-apoptotic agent in both rodent and human cell lines, and also can reduce chemically induced apoptosis and caspase-3 activation in the developing murine intestine [43]. A better understanding of gut-probiotic interactions may accelerate the development of novel therapeutic strains for human use.

## Conclusion

Our findings indicate that some of the tested bacteria simultaneously induced pro- and anti-inflammatory mediators and consequently helped to maintain a balance between the pro-inflammatory and regulatory cytokines, which is important for host immunity. Moreover, the tested strains able to survive in a host organism [12] exhibited comparable or better adhesion capacity in comparison to reference probiotic strains. According to these results, four strains (*Lb*. *plantarum* O20, *Lb*. *brevis* O22 and O24, and *Lb*. *rhamnosus* K3) exhibited immunomodulatory potential and can be selected for further studies. These newly identified lactobacilli hold promise for use as probiotics in functional food applications. Further studies will be required to better evaluate functional properties of tested strains.

## References

[1] Axelsson L (2004) Lactic acid bacteria: classification and physiology. In: Lactic acid bacteria microbiological and functional aspects, 3d edn. Marcel Dekker eds., pp 19–85

[2] de Vrese M, Schrezenmeir J (2008). Probiotics, prebiotics, and synbiotics. Food biotechnology.

[3] Walter J (2008). Ecological role of lactobacilli in the gastrointestinal tract: implications for fundamental and biomedical research. Appl Environ Microbiol.

[4] Wells JM (2011). Immunomodulatory mechanisms of lactobacilli. Microb Cell Facttories.

[5] Corthésy B, Gaskins HR, Mercenier A (2007). Cross-talk between probiotic bacteria and the host immune system. J Nutr.

[6] FAO/WHO (2002) Report of a joint FAO/WHO working group of drafting guidelines for the evaluation of probiotics in food: guidelines for the evaluation of probiotics in food, London

[7] Dong H, Rowland I, Tuohy KM, Thomas LV, Yagoob P (2010). Selective effects of *Lactobacillus casei* Shirota on T cell activation, natural killer cell activity and cytokine production. Clin Exp Immunol.

[8] Salminen S, Bouley C, Boutron-Ruault MC, Cummings JH, Franck A, Gibson GR, Isolauri E, Moreau MC, Roberfroid M, Rowland I (1998). Functional food science and gastrointestinal physiology and function. Br J Nutr.

[9] Isolauri E, Sütas Y, Kankaanpää P, Arvilommi H, Salminen S (2001). Probiotics: effects on immunity. Am J Clin Nutr.

[10] van Baarlen P, Wells JM, Kleerebezem M (2013). Regulation of intestinal homeostasis and immunity with probiotic lactobacilli. Trends Immunol.

[11] Sornplang P, Piyadeatsoontorn S (2016). Probiotic isolates from unconventional sources: a review. J Anim Sci Technol.

[12] Zielińska D, Rzepkowska A, Radawska A, Zieliński K (2015). *In vitro* screening of selected probiotic properties of *Lactobacillus* strains isolated from traditional fermented cabbage and cucumber. Curr Microbiol.

[13] Delgado S, O’Sullivan E, Fitzgerald G, Mayo B (2007). Subtractive screening for probiotic properties of *Lactobacillus* species from the human gastrointestinal tract in the search for new probiotics. J Food Sci.

[14] Chauviere G, Coconnier MH, Kernéis S, Fourniat J, Servin AL (1992). Adhesion of human *Lactobacillus acidophilus* strain LB to human enterocyte-like Caco-2 cells. Microbol.

[15] Crociani J, Grill JP, Huppert M, Ballongue J (1995). Adhesion of different bifidobacteria strains to human enterocyte-like Caco-2 cells and comparison with *in vivo* study. Lett Appl Microbiol.

[16] Ferreira V, Barbosa J, Stasiewicz M, Vongkamjan K, Switt AM, Hogg T, Gibbs P, Teixeira P, Wiedmann M (2011). Diverse geno-and phenotypes of persistent *Listeria monocytogenes* isolates from fermented meat sausage production facilities in Portugal. Appl Environ Microbiol.

[17] Tallon R, Arias S, Bressollier P, Urdaci MC (2007). Strain-and matrix-dependent adhesion of *Lactobacillus plantarum* is mediated by proteinaceous bacterial compounds. J Appl Microbiol.

[18] Laparra JM, Sanz Y (2009). Comparison of in vitro models to study bacterial adhesion to the intestinal epithelium. Lett Appl Microbiol.

[19] Jensen H, Grimmer S, Naterstad K, Axelsson L (2012). *In vitro* testing of commercial and potential probiotic lactic acid bacteria. Int J Food Microbiol.

[20] Tuomola EM, Salminen S (1998). Adhesion of some probiotic and dairy *Lactobacillus* strains to Caco-2 cell cultures. Int J Food Microbiol.

[21] Tulumoğlu Ş, Kaya Hİ, Şimşek Ö (2014). Probiotic characteristics of *Lactobacillus fermentum* strains isolated from tulum cheese. Anaerobe.

[22] Ramos CL, Thorsen L, Schwan RF, Jespersen L (2013). Strain-specific probiotics properties of *Lactobacillus fermentum*, *Lactobacillus plantarum* and *Lactobacillus brevis* isolates from Brazilian food products. Food Microbiol.

[23] Pisano MB, Viale S, Conti S, Fadda ME, Deplano M, Melis MP, Deiana M, Cosentino S (2014) Preliminary evaluation of probiotic properties of Lactobacillus strains isolated from Sardinian dairy products. BioMed Res Int, article ID 286390, 9 pages, doi:10.1155/2014/286390 10.1155/2014/286390 PMC4099116 25054135

[24] Schillinger U, Guigas C, Holzapfel WH (2005). *In vitro* adherence and other properties of lactobacilli used in probiotic yoghurt-like products. Int Dairy J.

[25] Glenting J, Beck HC, Vrang A, Riemann H, Ravn P, Hansen AM, Antonsson M, Ahrné S, Israelsen H, Madsen S (2013). Anchorless surface associated glycolytic enzymes from *Lactobacillus plantarum* 299v bind to epithelial cells and extracellular matrix proteins. Microbiol Res.

[26] Kos B, Suskovic J, Vukovic S, Simpraga M, Frece J, Matosic S (2003). Adhesion and aggregation ability of probiotic strain *Lactobacillus acidophilus* M92. J Appl Microbiol.

[27] Kotzamanidis C, Kourelis A, Litopoulou-Tzanetaki E, Tzanetakis N, Yiangou M (2010). Evaluation of adhesion capacity, cell surface traits and immunomodulatory activity of presumptive probiotic *Lactobacillus* strains. Int J Food Microbiol.

[28] Mathara JM, Schillinger U, Guigas C, Franz C, Kutima PM, Mbugua SK, Shin HK, Holzapfel WH (2008). Functional characteristics of *Lactobacillus* spp. from traditional Maasai fermented milk products in Kenya. Int J Food Microbiol.

[29] Tuo Y, Yu H, Ai L, Wu Z, Guo B, Chen W (2013). Aggregation and adhesion properties of 22 *Lactobacillus* strains. J Dairy Sci.

[30] Ren D, Li C, Qin Y, Yin R, Du S, Ye F, Liu C, Liu H, Wang M, Li Y, Sun Y, Li X, Tian M, Jin N (2014). *In vitro* evaluation of the probiotic and functional potential of *Lactobacillus* strains isolated from fermented food and human intestine. Anaerobe.

[31] von Martels JZ, Sadabad MS, Bourgonje AR, Blokzijl T, Dijkstra G, Faber KN, Harmsen HJ (2017). The role of gut microbiota in health and disease: in vitro modeling of host-microbe interactions at the aerobe-anaerobe interphase of the human gut. Anaerobe.

[32] Jensen H, Drømtorp SM, Axelsson L, Grimmer S (2015). Immunomodulation of monocytes by probiotic and selected lactic acid bacteria. Probiotics Antimicro Prot.

[33] Ménard S, Candalh C, Bambou JC, Terpend K, Cerf-Bensussan N, Heyman M (2004). Lactic acid bacteria secrete metabolites retaining anti-inflammatory properties after intestinal transport. Gut.

[34] Gaudana S, Dhanani A, Bagchi T (2010). Probiotic attributes of *Lactobacillus* strains isolated from food and of human origin. Br J Nutr.

[35] Gabay C (2006). Interleukin-6 and chronic inflammation. Arthritis Res Ther.

[36] Hooper LV, Wong MH, Thelin A, Hansson L, Falk PG, Gordon JI (2001). Molecular analysis of commensal host-microbial relationships in the intestine. Science.

[37] Howarth GS, Wang H (2013). Role of endogenous microbiota, probiotics and their biological products in human health. Nutrients.

[38] Aliprantis AO, Yang RB, Mark MR, Suggett S, Devaux B, Radolf JD, Klimpel GR, Godowski P, Zychlinsky A (1999). Cell activation and apoptosis by bacterial lipoproteins through toll-like receptor-2. Science.

[39] Li P, Ye X, Yang Q (2012). Antagonistic activity of *Lactobacillus acidophilus* ATCC 4356 S-layer protein on *Salmonella enterica* subsp. *enterica serovar* Typhimurium in Caco-2 cells. Ann Microbiol.

[40] Yan F, Polk DB (2002). Probiotic bacterium prevents cytokine-induced apoptosis in intestinal epithelial cells. J BiolChem.

[41] Yan F, Cao H, Cover TL, Whitehead R, Washington MK, Polk DB (2007). Soluble proteins produced by probiotic bacteria regulate intestinal epithelial cell survival and growth. Gastroenterology.

[42] Dykstra NS, Hyde L, MacKenzie A, Mack DR (2011). *Lactobacillus plantarum* 299v prevents caspase-dependent apoptosis in vitro. Probiotics Antimicrob Proteins.

[43] Lin PW, Nasr TR, Berardinelli AJ, Kumar A, Neish AS (2008). The probiotic *Lactobacillus* GG may augment intestinal host defense by regulating apoptosis and promoting cytoprotective responses in the developing murine gut. Pediatr Res.

